# Nitrogen and phosphorus co-doped carbon modified activated carbon as an efficient oxygen reduction catalyst for microbial fuel cells†

**DOI:** 10.1039/c7ra12907f

**Published:** 2018-01-03

**Authors:** Kang Lv, Hua Zhang, Shuiliang Chen

**Affiliations:** Department of Chemistry and Chemical Engineering, Jiangxi Normal University Nanchang 330022 China slchenjxnu@jxnu.edu.cn +86 791 8120740 +86 791 8120536

## Abstract

Activated carbon (AC) is an environmentally sustainable oxygen reduction reaction (ORR) catalyst and widely used in MFCs due to its intrinsic high specific surface area and mesoporous characteristics, but it shows relatively high ORR over-potential thus low electrocatalytic activity. In this study, a method of doped carbon modification was employed to decrease the over-potential and improve the ORR electrocatalytic activity of the AC catalyst. Nitrogen and phosphorus co-doped carbon modified AC (NPC@AC) was prepared by coating phytic acid doped polyaniline onto AC through *in situ* oxidative polymerization and subsequent high-temperature pyrolysis. The as-prepared NPC@AC possessed a large surface area of ∼649.3 m^2^ g^−1^ inherited from AC and a low ORR over-potential with a highly positive onset potential of +0.22 V *vs.* Ag/AgCl from NPC, thus showing an enhanced ORR electrocatalytic activity in neutral solution compared to the pristine AC, and even better than the pure NPC. The air-cathode MFC using the NPC@AC catalyst generated a much higher open circuit voltage of 0.753 V and two times higher power density of 1223 mW m^−2^ than that using the pristine AC catalyst of about 0.432 V and 595 mW m^−2^, respectively.

## Abstract 2

Nitrogen and phosphorus co-doped carbon modified activated carbon shows decreased ORR over-potential, thus enhanced ORR electrocatalytic activity in the air-cathode of microbial fuel cells compared to pristine AC.

## Graphical abstract

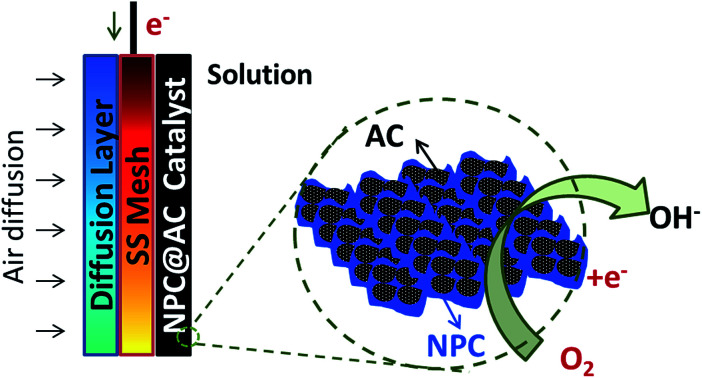

## 1. Introduction

Thanks to the dual-function of electricity generation and waste removal, microbial fuel cells (MFCs) have been targeted as a promising technology for the applications of renewable bioenergy, wastewater treatment, bio-sensing and bioremediation.^1–3^ The cathode is a crucial component of an MFC that determines its cost and performance. Notwithstanding, the oxygen reduction reaction (ORR) is the most important cathodic reaction in fuel cell technology (including MFCs) for power generation due to its high biochemical standard potential (+0.82 V *vs.* SHE) and its abundant availability from air. The most feasible cathode configuration for large-scale application of MFCs is the air-cathode because its use solves the issue of limited oxygen solubility in water and makes purging obsolete. The non-limiting availability of oxygen at air cathodes, ultimately, makes it possible to achieve higher current densities.^4–6^ Unfortunately, the sluggish ORR kinetics demands the use of efficient electrocatalysts. Pt-based catalysts are firstly used as the ORR catalyst in lab-scale MFC reactors.^7^ However, their high cost and poor stability hampering their use in practical applications of MFCs.^8,9^ Various alternative ORR catalysts to Pt have been proposed, including transition metal complexes,^10,11^ non-noble metal oxides,^12–15^ nitrogen-doped carbon nanotubes^16^ and graphene,^17^ and so on. Although these alternatives have been proved to be high efficient ORR catalysts for MFCs, the disadvantages, such as high manufacturing cost and complex fabrication procedure, hinder their practical application.

Activated carbon (AC) is a low-cost carbon material with large surface area (>500 m^2^ g^−1^) and has been widely used for adsorption, purification, catalyst and catalyst carrier.^18,19^ AC is also a cost-effective ORR catalyst for the air-cathode of MFCs.^20–24^ It has been reported that air-cathode using AC as ORR catalyst generated relatively higher power densities and showed better long-term durability than that using Pt/C catalyst. However, the electrocatalytic activity of the pristine AC toward ORR is unsatisfactory due to its high ORR over-potential. Direct doping of nitrogen is an effective strategy to improve the electrocatalytic activity of the AC for ORR.^25–28^ However, the improvement on the electrocatalytic activity of the AC is limited because a relatively low content of nitrogen (typically below 2%) can be incorporated to the AC. To further increase the content of N doping in the AC, Fe–N co-doped carbon had been incorporated to the AC and led to great increase of electrocatalytic performance of AC for ORR.^29,30^

It had been reported that introduction of a second heteroatom, such as B, S or P, to the N-doped carbon nanomaterials, was able to modulate the electronic and surface polarities, thus further increase the ORR catalyst activity of the carbon nanomaterial.^31–34^ At this point, those prior findings inspire us to explore a proper method on incorporated co-doped carbon to the AC to improve its ORR electrocatalytic activity. In this study, N and P co-doped carbon was incorporated to the AC (denoted as NPC@AC) by coating phytic acid doped polyaniline through *in situ* oxidative polymerization and subsequent high-temperature pyrolysis. The NPC@AC combined the good characteristics of high specific surface area of the AC and low ORR over-potential of the NPC, thus enhanced ORR electrocatalytic activity in neutral solutions comparing to the pure AC and NPC. MFC using the NPC@AC catalyst generated two times higher power density than that using the pristine AC catalyst.

## 2. Experimental

### 2.1 Preparation of NPC@AC

NPC@AC was prepared by a two-step process including phytic acid (PA) doped polyaniline coating and subsequent high-temperature pyrolysis. The coating of PA doped polyaniline onto AC was realized by *in situ* oxidative polymerization polyaniline in the presence of PA. In a typical procedure, 0.0625 mol ammonium persulfate was dissolved in 50 mL deionized water under stirring to prepare solution A. Solution B was prepared by mixing 50 mmol PA, 250 mmol aniline monomers in 100 mL of deionized water. Various amount of AC was dispersed in solution B. After both solutions being cooled down to 4 °C, solution A was poured into B and mixed swiftly by mechanical stirring, and kept under 4 °C for 8 hours without stirring. After washing with deionized water for three times to remove the excess acid and salts, the resultant PA doped carbon coated AC composite was dried and subsequently put into a tube furnace for pyrolysis. The pyrolysis process was conducted by heating to 950 °C with a temperature ramp of 5 °C min^−1^ in argon and annealing for 2 h at the final temperature. After naturally cooling to below 200 °C, the NPC@AC samples with NPC/AC ratio of 0.25, 0.4, 0.7 and 1.7 were obtained. These samples were denoted as NPC@AC-*x*, *x* represents the NPC/AC ratio. For comparison, the N mono-doped carbon and P mono-doped carbon modified AC were also prepared, and denoted as NC@AC and PC@AC, respectively. The NC@AC and PC@AC were prepared using the same procedures and same amount of reactants as that of the NPC@AC-0.7 sample, but using hydrochloric acid to replace the phytic acid when preparing NC@AC, and do not using aniline when preparing PC@AC.

### 2.2 Characterization and electrochemical test

The morphological characterization of the AC catalysts was conducted by using scanning electron microscope (SEM, Vega3, TESCAN) and transmission electron microscope (TEM, JEM-2100, JEOL). X-ray photoelectron spectroscopy (XPS, PHI Quantera SXM™) was introduced to exam the elemental composition and chemical state of samples. The crystalline structure was analyzed with powder X-ray diffraction (XRD, X'Pert-PRO) with a Cu K(alpha) target radiation source (*λ* = 0.154056 nm). The Raman spectra were recorded on a LabRAM Aramis (Horiba Jobin Yvon S.A.S) with a 633 nm wavelength laser.

All electrochemical measurements were conducted at a three-electrode system with 50 mM phosphate buffer solution (PBS, pH = 7.0) as electrolyte. Ag/AgCl (25 °C, saturated KCl internal solution) and Pt foil electrode were used as the reference and counter electrode, respectively. For cyclic voltammetry (CV) test, the catalysts were loaded onto a common glass carbon (*Φ* = 3 mm) and used as the working electrode. 1 mg catalyst was dispersed in a mixture solution containing 250 μL deionized water and 25 μL of 5 wt% Nafion® dispersion under sonication and formed a catalyst ink; then, 5 μL catalyst ink was pipetted onto a mirror-polished glassy carbon electrode and naturally dried up. For linear scanning voltammetry (LSV) tests, rotating disk electrode (RDE, *Φ* = 5 mm) and rotating ring-disk electrode (RRDE, *Φ* = 5 mm) were used. 15 μL catalyst ink was used for both RDE and RRDE. The LSV tests were conducted on the bi-potentiostat (Bio-logic, VMP3) equipped with a speed controller (AFMSRCE, Pine Instrument Co., USA). The electron transfer numbers (*n*) of catalysts were calculated according to the following equation:
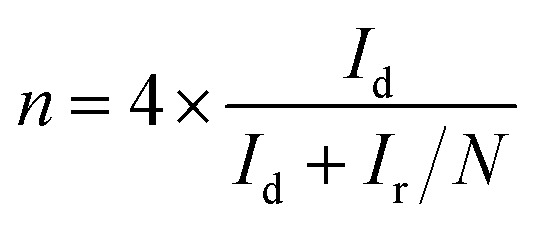
where *I*_d_ and *I*_r_ represent the disk and ring current, respectively. *N* refers to the collection efficiency of Pt ring and is taken as 40% in this study, which determined by the reduction of K_3_Fe[CN]_6_.

### 2.3 MFCs construction and operation

Single-chamber cubic air-cathode MFC set-up was used to measure the catalytic performance of the AC-based ORR catalysts in the MFC. The air-cathodes were fabricated by rolling method according to previous reports,^28,35^ using polyfluortetraethylene (PTFE) as binder and stainless steel mesh as current collector. The mass ratio of catalyst/PTFE was controlled to be about 4/1 and the loading of catalyst was controlled to be about 20 mg cm^−2^. Graphite fiber brushes (Jilin Chemical Fiber Group Co., Ltd., China) heat-treated at 450 °C for 30 min were used as anodes. A resistor with resistance of 1000 Ω was loaded between the brush anode and air-cathode. All MFCs were inoculated with effluent from another MFC, which was initially inoculated with wastewater from local wastewater treatment plant and operated for over 6 months. To keep the metabolism of bacteria, each reactor was fed with a medium containing acetate (1 g L^−1^), vitamin solution (12.5 mL L^−1^), trace element solution (12.5 mL L^−1^) in 50 mM phosphate buffer solution (PBS: pH 7.0, 10.9233 g L^−1^ Na_2_HPO_4_·12H_2_O, 3.042 g L^−1^ NaH_2_PO_4_·2H_2_O, 0.31 g L^−1^ NH_4_Cl, 0.13 g L^−1^ KCl). The medium was refreshed as soon as the voltage dropped below 100 mV. The cell voltage was monitored every minute by a date collection system (HIOKI LR8431-30). Polarization and power density curves were obtained by varying external circuit resistance with each resistor used for a full batch cycle. The power density and current density were normalized to the projected area of the air-cathode (7 cm^2^). Each measurement was repeated for at least three times.

## 3. Results and discussion

### 3.1 Electrocatalytic activity of NPC@AC with different NPC/AC ratio

**Fig. 1:**
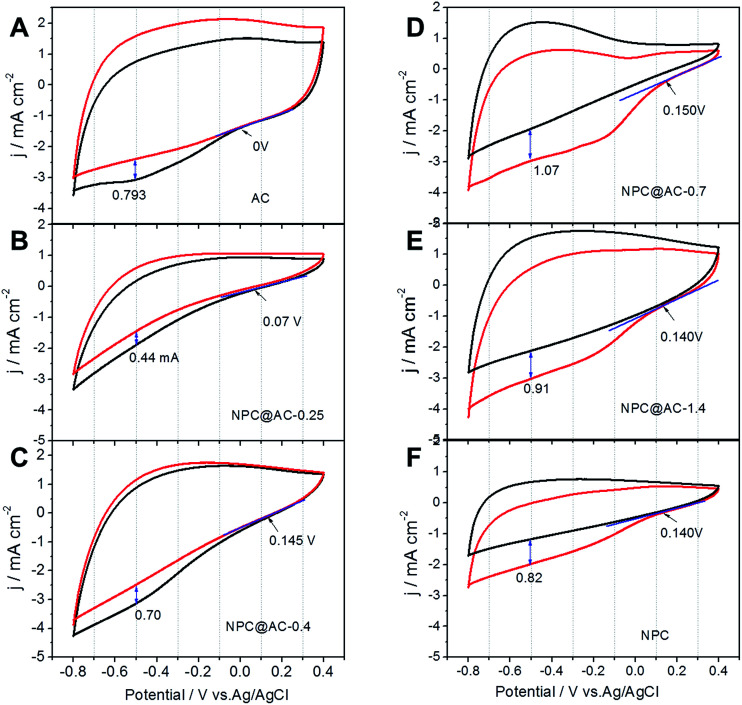
Cyclic voltammograms of NPC@AC electrocatalysts with different ratio of NPC/AC. (A) Pristine AC, (B) NPC@AC-0.25, (C) NPC@AC-0.4, (D) NPC@AC-0.7, (E) NPC@AC-1.4, (F) NPC, conducted in N_2_ (red color) and O_2_ (black color) saturated neutral 50 mM PBS. The scan rate is 100 mV s^−1^. The double-head arrows point out the ORR current densities at −0.5 V and the single-head arrows show the onset potentials.

The NPC@AC samples were fabricated by a two-step process including *in situ* oxidative polymerization and high-temperature pyrolysis. Here, aniline and PA were used as the nitrogen and phosphorus sources, respectively. In order to investigate the effect of NPC/AC ratio on the ORR electrocatalytic activity of the NPC@AC, cyclic voltammograms were performed in neutral media. As shown in Fig. 1, the NPC modification could bring great increase of ORR onset potential to the AC-based catalysts. The ORR onset potential of NPC@AC positively shift from 0 V of the pristine AC to +0.15 V of the NPC@AC-0.7, is kept at about +0.14 V with further increase the NPC content. Moreover, the NPC modification also leads to increase in the ORR current density. The NPC@AC-0.7 delivers a current density of 1.07 mA cm^−2^, which is 30% higher ORR current density than that of the pristine AC and even the pure NPC. In view of the best ORR electrocatalytic performance in terms of the more positive ORR onset potential and current density, thus the NPC@AC-0.7 were selected for further electrochemical performance investigation and compared to the single N doped (NC@AC) and P doped (PC@AC) carbon modified AC.

**Fig. 2:**
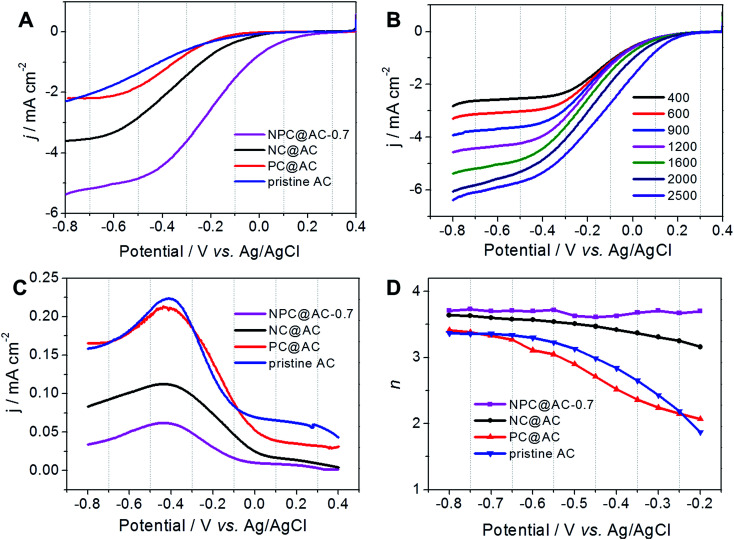
Electrocatalytic activity of the catalysts for ORR. (A) LSV curves of NPC@AC-0.7, NC@AC, PC@AC and pristine AC catalysts loaded on RDE at speed of 1600 rpm in O_2_-saturated neutral 50 mM PBS. (B) LSV curves of NPC@AC-0.7 in O_2_-saturated neutral 50 mM PBS at various rotating speeds. (C) Ring current response of different catalysts based on the RRDE measurements at a rotating speed of 1600 rpm in O_2_-saturated neutral 50 mM PBS. Scan rate is 10 mV s^−1^. (D) Electron transfer number (*n*) of different catalysts at different potentials.

RDE voltammograms were performed in O_2_-saturated 50 mM PBS and the results are shown in Fig. 2A. After doped carbon modification, the onset potential of the AC-based catalysts show a great positive shift from 0 V (pristine AC) to +0.22 V (NPC@AC-0.7), the ORR current densities also great increase, which is well in accordance to the CV results discussed in the preceding paragraph. Moreover, the ORR electrocatalytic activity of the NPC@AC-0.7 modified by co-doped carbon outperforms that of the NC@AC and PC@AC modified by mono-doped carbon. Furthermore, the RDE voltammograms of the NPC@AC-0.7 display a nearly one-step process of ORR at different rotating speeds (Fig. 2B), implying a four-electron ORR pathway. Moreover, RRDE measurements were conducted to study the generation of hydrogen peroxide for different catalysts (Fig. 2C). As expected, the NPC@AC-0.7 shows lower hydrogen peroxide generation than that of other catalysts. The average electron transfer numbers (*n*) of the NPC@AC-0.7 catalyst based on the RRDE measurements is about 3.6 (Fig. 2D). As can be seen, the NPC@AC-0.7 possesses excellent electrocatalytic activity for ORR in terms of positive ORR onset potential and high disc current, highlighting the importance of the N, P co-doping and mesoporous structure for ORR.

### 3.2 Morphological and elemental characterization

**Fig. 3:**
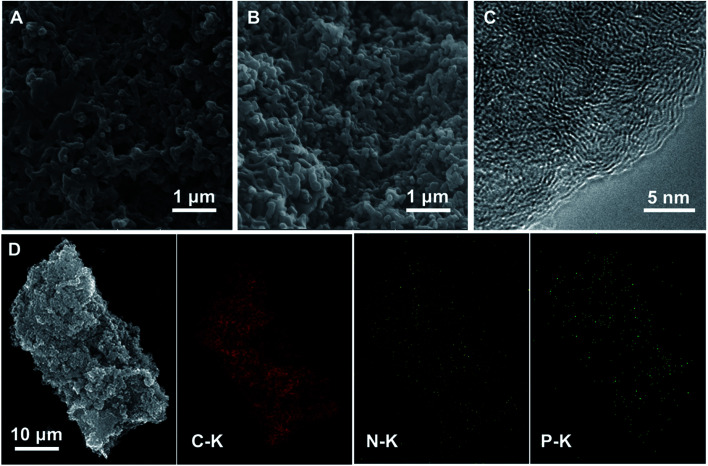
SEM images of the (A) PANi@AC and (B) NPC@AC. (C) High-resolution TEM image, (D) SEM and corresponding element mapping images of the NPC@AC-0.7. The element mapping images for C, N and P demonstrate a uniform distribution of the elements.

SEM morphological analyses demonstrate that the NPC@AC-0.7 exhibits a nanorod network surface (Fig. 3A and B) inherited from the PA doped PANi@AC. High-resolution transmission microscope (HR-TEM) image in Fig. 3C reveals that the NPC@AC-0.7 sample exhibits an edge-like graphitic carbon structure, which might be derived from of the NPC. To further detect the possible crystalline structure in the NPC@AC-0.7, XRD and Raman analyses were performed and the corresponding results are presented in Fig. S1.† XRD patterns in Fig. S1A† show that the pristine AC only has a board peak at around 23° corresponding to the (002) of the carbon. A new broad peak at around 43° corresponding to the (100) appears after incorporation of doped carbons, indicating the existence of graphitic carbon structure. Moreover, the intensity of the (100) NPC@AC-0.7 is stronger than that in the mono-doped carbon, NC@AC and PC@AC, demonstrating a higher average graphitization degree. This tendency could also be confirmed by the Raman analysis results. As shown in Fig. S1B,† the Raman spectra of the AC-based catalysts all display two wide peaks at ∼1327 cm^−1^ (D-band) and ∼1594 cm^−1^ (G-band). The intensity ratios of D band to G band (*I*_D_/*I*_G_) which reflects the average graphitization degree of different catalysts are calculated and summarized in Table 1. After doped carbon modification, the *I*_D_/*I*_G_ value of the AC-based catalysts decreased, demonstrating increase in the average graphitization degree. Moreover, the N and P co-doped NPC@AC-0.7 show lower *I*_D_/*I*_G_ value than mono-doped NC@AC and PC@AC, thus confirms the higher average graphitization degree in the NPC@AC-0.7, indicating the positive effect of phosphorus doping on the graphitization of the carbon layers.^36^

**Table 1:** Characteristics and textural parameters of AC-based catalysts

Samples	*S*_BET_ (m^2^ g^−1^)	*V*_pore_ (cm^3^ g^−1^)	*I*_D_/*I*_G_	Elemental analysis (at%)			
				C	N	O	P
NPC@AC-0.7	649.2	0.656	1.26	86.87	4.54	6.37	2.21
NC@AC	500.6	0.514	1.42	92.61	4.60	2.80	—
PC@AC	456.0	0.364	1.52	88.29	0.55	7.57	3.59
Pristine AC	943.5	0.919	1.64	92.53	1.22	6.24	—

**Fig. 4:**
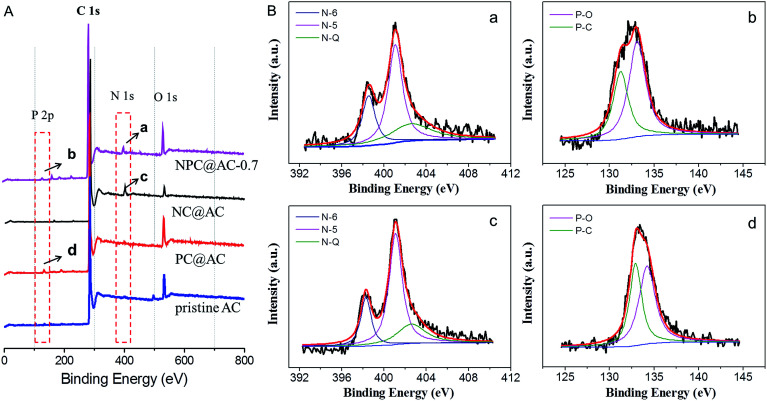
XPS spectra of catalyst samples (A) survey and (B) high-resolution XPS spectra of the selected peaks in A, (a) N 1s and (b) P 2p fine spectra of NPC@AC-0.7; (c) N 1s fine spectrum of NC@AC and (d) P 2p fine spectrum of PC@AC, respectively.

To confirm the incorporation of heteroatom (N and/or P) doped carbons to the AC, XPS analysis was conducted and the spectra are shown in Fig. 4A, and the textural parameters are summarized in Table 1. As expected, the XPS spectrum of confirms the presence of the O, N, and P heteroatom in the NPC@AC-0.7 with N and P content of 4.54% and 2.21%, respectively. The fine N 1s spectra of the NPC@AC-0.7 (Fig. 4Ba) and NC@AC (Fig. 4Bc) can be fitted into three bands at ∼398.6, 401.3 and 402.5 eV, which correspond to pyridinic (N-6), pyrrolic (N-5) nitrogen and pyridine-*N*-oxide groups (N-Q), respectively.^37–39^ The incorporation of N atom to carbon ring could change the spin density and atomic charge distribution of carbon atoms in the graphitic layers, which effectively weakened the O–O bonding energy, thus facilitated ORR catalytic activity.^40,41^ The fine P 2p spectra of the NPC@AC (Fig. 4Bb) and PC@AC (Fig. 4Bd) can be fitted into two bands at ∼131.8 and 133.6 eV, which were ascribed to P–C and P–O states, respectively. It was reported that P-doping could enhance the charge delocalization of the carbon atoms and produce carbon structures with many edge sites.^42^ These electrical and physical alternations of carbon substrate caused by N, P co-doping would be more favorable for the reduction of the oxygen on the carbon surface, thus probably give rise to high ORR catalytic activity.

N_2_ adsorption–desorption isotherm curves (Fig. S2A†) show that the NPC@AC-0.7 preserve a high specific surface area of 649.2 m^2^ g^−1^ and a total pore volume of 0.656 cm^3^ g^−1^ (Table 1), which is about 70% specific surface area and pore volume of the pristine AC (943.5 m^2^ g^−1^ and 0.919 cm^3^ g^−1^). The relatively lower specific surface area the NPC@AC-0.7 is likely due to the occupancy of partial pores of the AC by the deposition of NPC during pyrolysis process. Barrett–Joyner–Halenda (BJH) pore size distribution curves of the as-prepared catalysts (Fig. S2B†) confirm the presence of mesopores with diameters <10 nm, which would be in favor for electrocatalytic applications.

### 3.3 Performance of the electrocatalyst in the air-cathode of MFCs

**Fig. 5:**
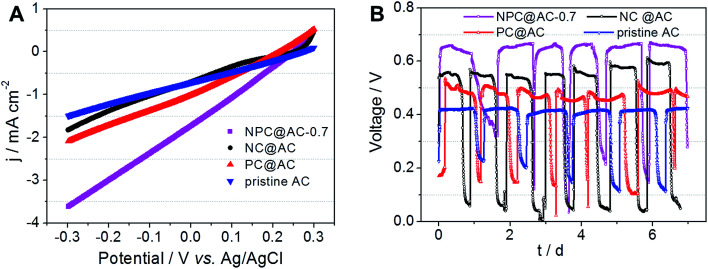
(A) LSV and (B) *V*–*t* curves of MFC equipped with air-cathodes using catalysts of NPC@AC, NC@AC, PC@AC and pristine AC. The media is 50 mM PBS (pH = 7.0) containing 1 g L^−1^ acetate. Scan rate is 1 mV s^−1^.

The practical performance of different catalysts in MFCs, air-cathodes with various ORR catalysts were prepared by rolling method as reported previously.^28,35^ The LSV curves in Fig. 5A demonstrate that all the air-cathodes with doped carbon modified AC catalysts exhibit higher current response than that of pristine AC catalyst. Comparing to the NC@AC and PC@AC, the NPC@AC-0.7 displays the highest current density. As shown in Fig. 5B, air-cathode MFC using NPC@AC-0.7 ORR catalyst is able to generate a stable voltage of 0.668 V across an external resistor of 1000 Ω, which is much higher than that of using the NC@AC (0.583 V), PC@AC (0.517 V) and pristine AC (0.432 V) catalysts, respectively. These results imply that modification by doped carbon is an effective method to enhance the ORR activity of the AC. Moreover, the NPC@AC-0.7 prepared by N and P co-doped carbon modification outperforms the NC@AC and PC@AC by mono-doped carbon modification, when using as ORR catalyst in the air-cathode of MFC.

**Fig. 6:**
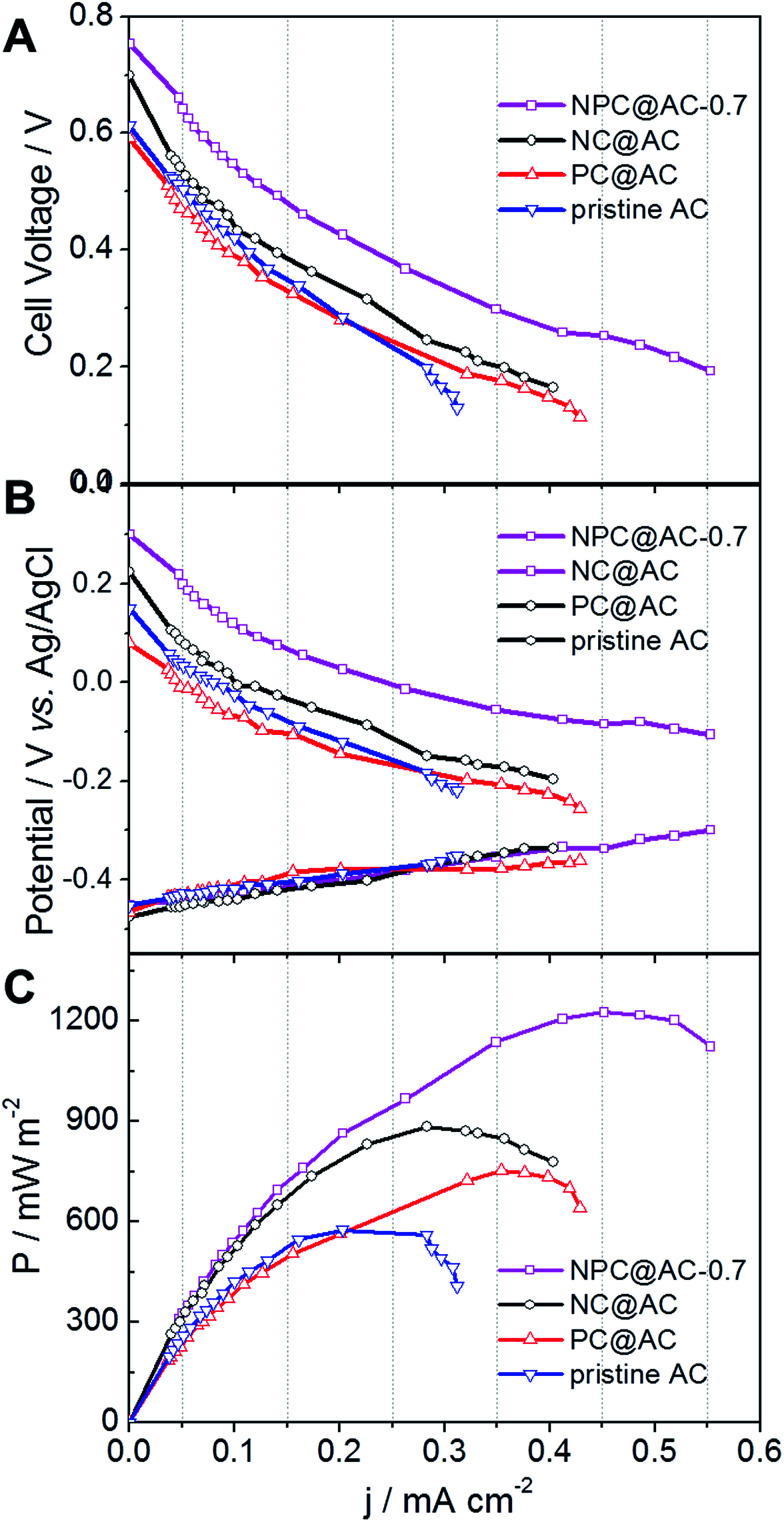
(A) Cell and (B) electrode polarization and (C) power density curves of air-cathode MFCs using different AC based catalysts.

As shown in Fig. 6A, using the NPC@AC-0.7 catalyst, the air-cathode MFC is able to generate a higher open circuit voltage (OCV) of 0.753 V than that of using other catalysts. Moreover, the electrode polarization curves in Fig. 6B show that the increase of OCV is mainly brought by the improvement of performance at the air-cathode. These results demonstrate that incorporation of NPC to the AC greatly reduce the over-potential of air-cathode towards ORR, thus lead to high OCV. The power density curves of air-cathode MFC using different catalysts in Fig. 6A reveal that the air-cathode MFC using the NPC@AC-0.7 ORR catalyst shows better performance than the mono-doped carbon modified AC, NC@AC and PC@AC, and the pristine AC. And the power densities arranged in this order: pristine AC (595 mW m^−2^) < PC@AC (762 mW m^−2^) < NC@AC (895 mW m^−2^) < NPC@AC-0.7 (1223 mW m^−2^). The power density from the MFC with NPC@AC-0.7 catalyst (1223 mW m^−2^) is two times higher than that with pristine AC. These results demonstrate that the NPC@AC-0.7 is an efficient ORR catalyst for air-cathode of MFCs.

## 4. Conclusions

The method of doped carbon modification was employed to improve the ORR electrocatalytic activity of the AC electrocatalyst. The doped carbon modification were realized by using a facilely and scalable process including steps of coating of PA doped polyaniline by *in situ* oxidative polymerization and subsequent high-temperature pyrolysis. The NPC@AC-0.7 modified by N and P co-doped carbon, inherited the large surface area and mesoporous characteristics from the AC and had improved degree of graphitization, thus exhibited higher ORR electrocatalytic activity in term of both the onset potential and current density in neutral solution, comparing to the mono-doped carbon modified AC, NC@AC and PC@AC, and the pristine AC. The MFC using the NPC@AC-0.7 as ORR catalyst generated a much higher open circuit voltage of 0.753 V and two times higher power density of 1223 mW m^−2^, comparing to the MFC using the pristine AC ORR catalyst. The NPC@AC-0.7 would be a low-cost and efficient ORR catalyst for practical applications of MFCs.

## Conflicts of interest

There are no conflicts to declare.

## Supplementary Material

RA-008-C7RA12907F-s001
