# Supplementary material for: Nitrogen and phosphorus co-doped carbon modified activated carbon as an efficient oxygen reduction catalyst for microbial fuel cells
Source: RSC Adv. 2018 Jan 3;8(2):848–55. doi: 10.1039/c7ra12907f (PMC9076873; doi:10.1039/c7ra12907f)
Supplement: RA-008-C7RA12907F-s001 [file RA-008-C7RA12907F-s001.pdf]

Electronic Supplementary Material (ESI) for RSC Advances.
This journal is © The Royal Society of Chemistry 2017

Nitrogen and Phosphorus co-doped Carbon Modified Activated Carbon as Efficient Oxygen Reduction Catalysts for Microbial Fuel Cells

Kang Lv, Hua Zhang and Shuiliang Chen*

Department of Chemistry and Chemical Engineering, Jiangxi Normal University, 330022 Nanchang, China. E-mail: slchenjxnu@jxnu.edu.cn

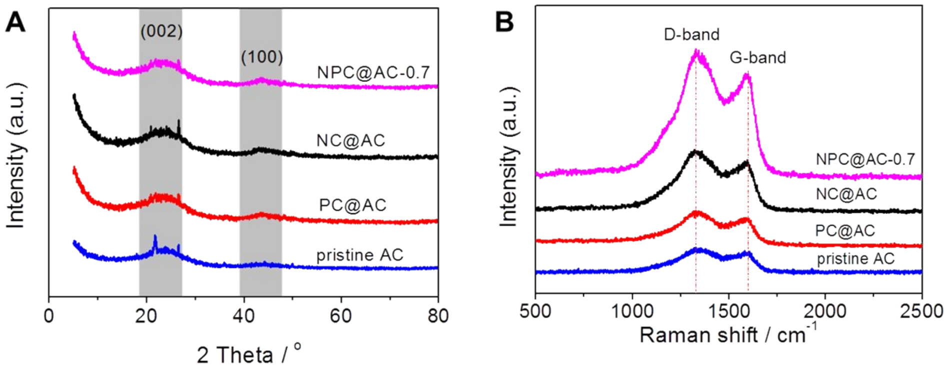

Fig. S1. (A) XRD patterns and (B) Raman spectra of the NPC@AC-0.7, NC@AC, PC@AC and pristine AC.

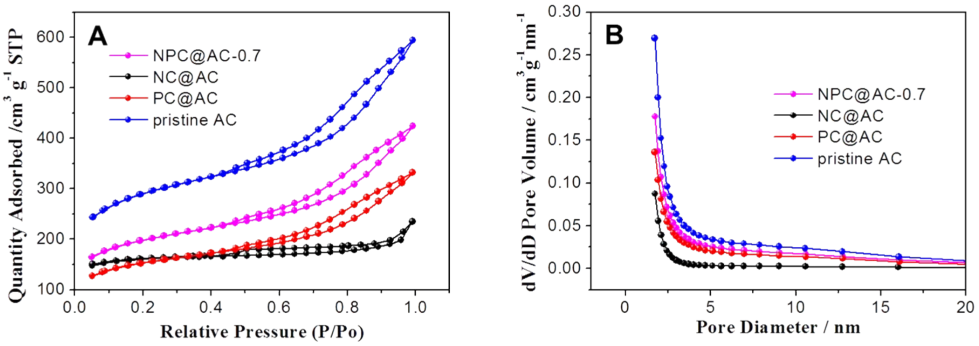

Fig. S2. (A) N_2 adsorption–desorption isotherms and (B) pore size distributions of the NPC@AC-0.7, NC@AC, PC@AC and pristine AC, respectively.
